# Expression of the ARPC4 Subunit of Human Arp2/3 Severely Affects *Mycobacterium tuberculosis* Growth and Suppresses Immunogenic Response in Murine Macrophages

**DOI:** 10.1371/journal.pone.0069949

**Published:** 2013-07-22

**Authors:** Anamika Ghosh, Sultan Tousif, Debapriya Bhattacharya, Sachin K. Samuchiwal, Kuhulika Bhalla, Megha Tharad, Sushil Kumar, Prem Prakash, Purnima Kumar, Gobardhan Das, Anand Ranganathan

**Affiliations:** 1 Recombinant Gene Products Group, International Centre for Genetic Engineering and Biotechnology, ICGEB Campus, Aruna Asaf Ali Marg, New Delhi, India; 2 Immunology Group, International Centre for Genetic Engineering and Biotechnology, ICGEB Campus, Aruna Asaf Ali Marg, New Delhi, India; 3 Department of Biochemistry, University of Calcutta, Kolkata, India; 4 Kwazulu-Natal Research Institute for tuberculosis and HIV, University of Kwazulu-Natal, Durban, South Africa; Tulane University, United States of America

## Abstract

**Background:**

The search for molecules against *Mycobacterium tuberculosis* is urgent. The mechanisms facilitating the intra-macrophage survival of *Mycobacterium tuberculosis* are as yet not entirely understood. However, there is evidence showing the involvement of host cell cytoskeleton in every step of establishment and persistence of mycobacterial infection.

**Methodology/Principal Findings:**

Here we show that expression of ARPC4, a subunit of the Actin related protein 2/3 (Arp2/3) protein complex, severely affects the pathogen’s growth. TEM studies display shedding of the mycobacterial outer-coat. Furthermore, in infected macrophages, mycobacteria expressing ARPC4 were cleared off at a much faster rate, and were unable to mount a pro-inflammatory cytokine response. The translocation of ARPC4-expressing mycobacteria to the lysosome of the infected macrophage was also impaired. Additionally, the ARPC4 subunit was shown to interact with Rv1626, an essential secretory mycobacterial protein. Real-time PCR analysis showed that upon expression of ARPC4 in mycobacteria, Rv1626 expression is downregulated as much as six-fold. Rv1626 was found to also interact with mammalian cytoskeleton protein, Arp2/3, and enhance the rate of actin polymerization.

**Conclusions/Significance:**

With crystal structures for Rv1626 and ARPC4 subunit already known, our finding lays out the effect of a novel molecule on mycobacteria, and represents a viable starting point for developing potent peptidomimetics.

## Introduction

About one third of the world population is latently infected with *Mycobacterium tuberculosis* (Mtb). There has been no new drug against Mtb for more than four decades, although recent discoveries of small molecules show promise [1], [2]. Knowledge of the exact mycobacterial target protein for a particular drug is nowadays considered crucial for understanding the mechanism of action of anti-TB moieties. Moreover, the rapid spread of drug-resistant Mtb has necessitated the requirement of target information. However, discovery of new anti-TB molecules being a slow and cumbersome process, a variety of strategies need to be employed. One strategy is to use proteins and peptide libraries as a starting point to discover entities that bind to specific Mtb targets. Hits discovered this way can either be used on their own, or as a template for discovering potent peptidomimetics. In a related field, numerous peptidomimetic inhibitors of the Hep C protease have been discovered and two among them, Telaprevir and Bocepravir, have recently entered the market [3], [4].

As an ongoing effort to pursue such a strategy, we report here the discovery, that a known human protein, the ARPC4 subunit of the human Arp2/3 complex, severely affects Mtb growth and shows significant alterations in immune response *ex vivo*. Employing the rational approach means that the Mtb target of ARPC4 is known as well. It is the essential protein Rv1626, the knowledge of the structure of which has linked it to several putative functions.

Once the bacillus finds its niche in a macrophage, it secretes proteins in its milieu to make the environment habitable. Rv1626 is one such secretory protein that has been predicted to perform the role of a Two-Component System (TCS) response regulator, with Rv3220c as its cognate histidine kinase [5], [6]. Both these proteins are conserved across all mycobacterial species and exhibit strong expression after 18 hours of intracellular growth that starts declining after 110 hours, suggesting that these proteins are important during the early stages of Mtb growth [7], [8]. Rv1626 also shows high structural similarity to a known transcription anti-termination factor, AmiR from *Pseudomonas aeruginosa* [9].

Rv1626 is enlisted in Rubin’s list of essential proteins of Mtb but its functions are still a matter of speculation [10]. As Rv1626 is constitutively synthesized and secreted out by the bacterium [5], [8], [11], [12], some of its functions should rationally also be extracellular. Indeed, in a comprehensive and seminal study Belisle and co-workers showed Rv1626 to be one of the hundred odd proteins that constitute the secretory proteome of Mtb, as they were able to purify Rv1626 from the culture filtrate using a combination of anion exchange chromatography and SDS-PAGE, followed by electroelution [11].

We found that the ARPC4 subunit of the mammalian cytoskeletal protein Arp2/3 interacts with Rv1626, and the expression of ARPC4 inside mycobacteria causes severe effects that could be because of this interaction. Intriguingly, we find that, like other Arp2/3 interacting pathogenic proteins [13]–[15], Rv1626 enhances the actin polymerization efficacy of Arp2/3.

We speculate on the possibility of the interaction between mycobacterial Rv1626 and mammalian Arp2/3 being of the same significance as it is for other intracellular pathogens that synthesize proteins that directly or indirectly modulate host cell cytoskeleton for their survival and perpetuation. Also, as ARPC4 expression inside Mtb causes significant deleterious effects on its survival and infectivity, structural analogues of ARPC4 can be explored for development of anti-TB drug candidates.

## Materials and Methods

### *In vivo* Protein-Protein Interaction: Bacterial Two-Hybrid Studies

Bacteriomatch™ two-hybrid system kit and human lung cDNA library (cloned in pTRG vector) were purchased from Stratagene, USA. The full length *rv1626* gene was PCR amplified from Mtb H37Rv genomic DNA using forward and reverse primers (Table 1) and following subcloning into pGEMT easy vector, was cloned in modified pBT vector, pBTnn [16].

**Table 1:** Sequences of the DNA primers used for PCR amplification of various genes described in the present study.

Primer Name	Primer Sequence
*ARPC4* forward	5′-CCG AAT TCT ACG TAA TGA CTG CCA CTC TCC GCC CCT ACC TG-3′
*ARPC4* reverse	5′-CCG AAT TCT ACG TAA AAA TTC TTA AGG AAC TCT TCA GCC ACA A-3′
*rv1626* forward	5′-CCT ACG TAA TGA CCG GCC CCA CCA CCG ACG CCG A-3′
*rv1626* reverse	5′-CCT ACG TAG GTG TCT TTG GGT GTT CCG AGG GTT-3′
*bfrB* forward	5′-ACA GAA TAC GAA GGG CCT AA-3′
*bfrB* reverse	5′-ACG AAG GTC GCG GTC GAG CA-3′
*16S rrna* forward	5′-GCA CCG GCC AAC TAC GTG-3′
*16S rrna* reverse	5′-GAA CAA CGC GAC AAA CCA CC-3′

The reporter *E. coli* strain was co-transformed with equal amounts (250 ng each) of Rv1626-pBTnn and human lung cDNA library and plated on X-Gal indicator plates containing kanamycin (50 µg/ml), chloramphenicol (30 µg/ml), tetracycline (12.5 µg/ml), X-Gal (80 µg/ml), Isopropyl β-D-1-thiogalactopyranoside, IPTG (25 µM), and phenylethyl β-D-thiogalactoside (200 µM). Plasmids pBT-LGF2, pTRG-Gal11p (manufacturer provided positive controls) and empty pBTnn plasmid (for negative control) were co-transformed in proper combinations. Positive interactions were judged by the blue colour of the colonies obtained and further verified by repeated clonings and co-transformations. All interactions were further verified by liquid β-galactosidase assay performed as described earlier [17] and the statistical significance of the interactions was evaluated by Student’s t-test.

### Cloning of *ARPC4* Gene

Full length *ARPC4* gene was re-cloned into modified pTRG vector, pTRGnn [16]. The gene was amplified from ARPC4pTRG (fished out from the lung cDNA library) using forward and reverse primers (Table 1), the PCR product was *Sna*BI digested, and was ligated in *Sna*BI-cut pTRGnn plasmid.

### Cloning of Rv1626 and ARPC4 for Protein Expression

In a manner similar to above, *rv1626* gene was PCR-amplified (primer details in Table 1), *Sna*BI-digested and ligated into *Sna*BI-cut Stop-pET28 and pGEX4T3nn vectors [16]. These vectors, upon induction, produce tag-less Rv1626 protein and a fusion protein of Rv1626 with an N-terminal GST tag, respectively. Also, *Sna*BI-digested *ARPC4* PCR product was cloned into *Sna*BI-digested Bla1cut-pET28a [16]. This vector, upon induction, produces ARPC4 protein with a C-terminal hexa-Histidine tag (ARPC4-His_6x_).

### Protein Expression and Purification of ARPC4-His_6x_

Exponentially growing *E. coli* BL21 (DE3) cells harbouring ARPC4Bla1cut-pET28a were induced with 1 mM IPTG for 3 hours at 37°C. Harvested cell pellet was washed with PBS (137 mM NaCl, 2.7 mM KCl, 10 mM NaH_2_PO_4_ and 2 mM K_2_HPO_4_, pH 7.4), resuspended in lysis buffer (6 M Guanidine hydrochloride, 10 mM Tris, 100 mM sodium phosphate buffer, 150 mM NaCl, 0.1% Tween-20 and 0.01% CHAPS, pH 7.2), and lysed by sonication. Clear cell lysate was incubated with lysis buffer-equilibrated Qiagen Ni-NTA agarose beads for 2 hours and protein was purified pH-based elution at room temperature. Column was washed with lysis buffer, wash buffer 1 (10 mM Tris, 100 mM sodium phosphate buffer, 50 mM NaCl, 8 M Urea, pH 6.3) and wash buffer 2 (10 mM Tris, 100 mM sodium phosphate buffer, 8 M Urea, pH 5.9). The resin bound proteins were eluted with elution buffer (10 mM Tris, 100 mM sodium phosphate buffer, 8 M Urea, pH 4.5) and dialyzed against storage buffer (50 mM L-glutamate, 50 mM L-arginine in 20 mM sodium acetate buffer, pH 5.0) to remove urea and was stored at −20°C till further use.

### Protein Expression and Purification of Rv1626

*E. coli* BL21 (DE3) (Novagen) cells, harbouring Rv1626-Stop pET28 vector, were grown till mid-log phase and induced with 1 mM IPTG for 3 hours with constant shaking at 37°C. Cells were harvested and the pellet was washed with PBS buffer. Cell pellet was resuspended in (1% of the original culture volume) lysis buffer (4 mM dithiothretol, 20 mM Tris-HCl pH 8.0) that was supplemented with 2 mM PMSF and sonicated till a clear lysate was obtained. This lysate was then centrifuged to obtain a clear supernatant that was transferred into fresh tubes. Rv1626 protein was purified by the method described earlier [9]. Briefly, the clear lysate was run through a 5.0 ml HiTrap Q HP column (GE Healthcare Life Sciences) equilibrated in Buffer A (50 mM KCl, 2 mM dithiothretol, 20 mM Tris-HCl pH 8.0) and a 20 column volume continuous gradient was run from Buffer to Buffer B (1 M KCl, 2 mM dithiothretol, 20 mM Tris-HCl pH 8.0) using the GE Healthcare Life Sciences Pharmacia Biotech AKTA FPLC pumping system. Rv1626 protein eluted at around 400 mM KCl. Relevant fractions were pooled and concentrated to 2 ml volume using Vivaspin 15 centrifugal concentrator (VivaProducts). The protein was further purified by gel permeation chromatography using a HiLoad 16/60 Superdex 75 pg column (GE Healthcare Life Sciences) equilibrated in buffer A. Peak fractions were pooled, dialyzed against PBS and stored at −20°C in aliquots till further use.

### Protein Expression and Purification of GST-Rv1626

Exponentially growing *E. coli* BL21 (DE3) (Novagen) cells harbouring Rv1626-pGEX4T3nn were induced with 1 mM IPTG for 16 hours at 18°C. Harvested cells were washed with PBS buffer, resuspended in PBS buffer supplemented with 2 mM PMSF (phenylmethanesufonyl fluoride) and sonicated till a clear lysate was obtained. Clear cell lysate was incubated with PBS-equilibrated Glutathione Sepharose-4B beads (GE Healthcare Life Sciences) at 4°C overnight. Following PBS washes, resin bound proteins were eluted using elution buffer (10 mM reduced glutathione in 50 mM Tris-Cl, pH 8.0) and dialyzed against PBS and stored at −20°C in aliquots till further use.

### *In vitro* Protein-Protein Interaction

Protein-protein interaction *in vitro* was confirmed by dot Far-Western Blot analyses and were performed according to protocols described previously [18], [19].

#### Rv1626 and ARPC4

Purified ARPC4-His_6x_, GST and GST-Rv1626 proteins were immobilized on four strips of nitrocellulose membrane (1 µg protein per spot). The strips were incubated in blocking solution, 5% non-fat dry milk and 2% polyvinyl pyrrolidone (PVP) in PBS-T buffer (1% Tween-20 in PBS), for 2 hours at room temperature. After three washes with PBS-T, strips (a) and (c) were incubated overnight in 1 µg/ml solutions of GST-Rv1626 and GST respectively in cold room with constant shaking. 1 µg/ml protein solutions were prepared in a solution of composition 1% PVP and 2.5% non-fat dry milk in PBS-T. The strips were then probed with anti-GST antibodies (Sigma). The control strips, (b) and (d), were directly probed with anti-pentaHis (Qiagen) and anti-GST antibodies, respectively. HRPO-conjugated secondary antibodies (goat anti-mouse IgG) were purchased from Calbiochem and all the strips were developed using TMB (Sigma) as substrate.

#### Rv1626 and Arp2/3

Arp2/3 (Cytoskeleton Inc., USA) and BSA (Sigma) proteins were immobilized on two strips of nitrocellulose membrane (1 µg protein per spot). The strips were incubated in blocking solution for 2 hours at room temperature as above. After three washes with PBS-T, the strips (a) and (b) were incubated overnight in 1 µg/ml solution of GST and GST-Rv1626, respectively in cold room with constant shaking. Protein solutions were made just as above. The strips were subsequently probed with anti-GST antibodies, followed by HRPO-conjugated secondary antibodies and developed with TMB.

### Actin Polymerization Assay

Actin polymerization kit was acquired from Cytoskeleton Inc., USA, and the assay was performed as per the instruction manual and as described earlier [15]. Briefly, actin polymerization was monitored as enhanced pyrenyl-actin fluorescence. Pyrene-labelled rabbit muscle actin in G-buffer (0.2 mM CaCl_2_, 0.2 mM ATP, 5 mM Tris-HCl pH 8.0) was pre-cleared by centrifugation. To this pre-cleared G-actin (3.6 µM, pyrene-labelled), Rv1626 (500 nM), VCA domain (400 nM; Cytoskeleton Inc.), an unrelated protein (500 nM) and Arp2/3 (10 nM) alone or in the combinations were added and the baseline was stabilized before initiating the polymerization by addition of the actin polymerization buffer (50 mM KCl, 2 mM MgCl_2_, 1 mM ATP, 0.5 mM dithiothretol, 1 mM EGTA, 0.1 mM CaCl_2_ 10 mM Tris-HCl pH 7.5). Fluorescence measurements were made with Molecular Devices SpectraMax M2 Spectrofluorimeter at 25°C, with excitation and emission wavelengths set at 350 and 407 nm respectively.

### Cloning of ARPC4 in Mycobacterial Expression Vector pVV16

The *ARPC4* gene was amplified from original lung cDNA library clone, and the *Sna*BI digested PCR product was cloned into *Sna*BI-digested Mtb shuttle vector pVV16 to generate ARPC4pVV16 [20]. One of the positive clones was used for further transformation in Mtb H37Rv by electroporation. Positive mycobacterial clones were identified by PCR following plasmid DNA extraction from Mtb.

### Confirmation of *ARPC4* Gene Expression in H37Rv

The expression of ARPC4 protein in H37Rv cells was confirmed by checking the synthesis of *ARPC4* gene transcript. For this, total RNA was extracted from exponentially growing cultures of Mtb H37Rv (control) and H37Rv harbouring the ARPC4pVV16 plasmid (H37Rv/ARPC4) and reverse transcribed to obtain cDNA (as per the methods described below). This cDNA was PCR amplified using ARPC4 forward and reverse primers and the product was checked on 1% agarose gel.

### RNA Extraction from Mtb

Total RNA was extracted as described previously [20].

### Growth Curve Studies of Mtb

Mtb harboring ARPC4pVV16 (H37Rv/ARPC4) or pVV16 (H37Rv/pVV16) vectors were grown in 10 ml Middlebrook 7H9 broth (Difco™, BD) supplemented with 10% ADC (BBL™, BD), 0.05% Tween-80, 0.2% glycerol, and kanamycin (25 µg/ml) at 37°C and constant shaking till stationary phase was reached. Optical density of the culture was measured at 600 nm using Lambda 35 spectrometer (PerkinElmer) and equal numbers of cells from each culture were freshly inoculated in 30 ml flask containing fresh 7H9 kanamycin broth such that the initial O.D. of the culture is 0.05. Cells were allowed to grow at 37°C with constant shaking. Optical density of each culture was measured and recorded after every 24 hours.

### CFU Count

To corroborate the growth curve results a Colony Forming Units or CFU count was performed. At time-points of 0, 7 and 14 days, four dilutions (made in PBS) of the H37Rv/ARPC4 and H37Rv/pVV16 cultures, being grown and monitored for growth curve analyses, were plated on 7H11 agar (Difco™, BD) plates supplemented with 0.5% glycerol and 10% OADC (BBL™, BD), such that the highest dilution would give approximately between 10–100 colonies. The plates were incubated at 37°C for 15 days before the number of colonies were counted and recorded.

### Transmission Electron Microscopy

Mtb H37Rv cells and H37Rv cells harboring ARPC4pVV16 were grown at 37°C with constant shaking till mid-log phase. Cells were harvested and fixed with 2% paraformaldehyde solution as described earlier [21], were allowed to adsorb on a carbon coated 300 square mesh copper grid (Electron Microscopy Sciences, USA) and air dried. Samples were then stained with 1% uranyl acetate for 30 seconds and photographed using a FEI Tecnai 12 Electron Microscope.

### Real Time PCR

100 ng RNA from control (H37Rv) and test (H37Rv/ARPC4) samples was reverse transcribed using RevertAid™ H minus first strand cDNA synthesis kit (Fermentas) with random hexamer primers in a total volume of 10 µl. The reaction mixture was incubated in a thermocycler at 25°C for 5 minutes, 42°C for 60 minutes, 85°C for 5 minutes and finally cooled to 4°C.

Real time PCR was performed using Maxima SYBR Green/Flourescein qPCR master mix (Fermentas) in a total reaction volume of 25 µl consisting of 0.3 µM of each primer (forward and reverse primers of Rv1626, BfrB and 16S rRNA genes; Table 1), 12.5 µl of 2× Maxima SYBR Green/Flourescein qPCR master mix, and 5 µl of diluted cDNA template (equivalent to 5 ng RNA). Amplification was performed in a MiniOpticon (BioRad) thermocycler in the following conditions: (i) an initial denaturation step of 3 minutes at 95°C; (ii) 40 cycles, each consisting of 30 seconds at 95°C, 30 seconds at 60°C, and 30 seconds at 72°C. Fluorescence was measured at the end of the elongation step of each cycle. A melt curve analysis was performed between 55°C and 95°C with an increment of 0.5°C per 30 seconds. PCR products were also checked on 1% agarose gel. Data was analyzed using comparative Ct method as described earlier [22]. *16S rrna* was used as an internal control gene for normalization and an unrelated *bfrB* gene was included as a negative control.

### Murine Macrophage Infection Studies

#### Mice

C57BL/6 male mice at 6–8 weeks of age were used throughout the study following institutional ethical committee guidelines. All animal experiments were conducted in accordance with guidelines approved by the Institutional Animals Ethics Committee of ICGEB, New Delhi, India and Department of Biotechnology (DBT), Government of India. Mice were housed under barrier conditions in a Biosafety Level III laboratory.

### CFU Counts Post-infection

2.5 ml of autoclaved thioglycolate was given to 6 to 7 weeks old male C57BL/6 mice and kept for 4 days in pathogen free environment. On day 5, they were sacrificed and intraperitoneal macrophages were isolated. Macrophages were counted and 1×10^6^ cells were seeded in 12 well plates (Nunc, USA) with 10% RPMI 1640 (Gibco, Invitrogen, UK). Cultured cells were kept for 24 hours and then infected with exponentially growing culture of Mtb H37RV and Mtb H37RV/ARPC4 at an MOI of 10. At specific time-points (0, 24, 48 and 72 hrs), the macrophages were lysed using 0.04% SDS in 7H9 medium and four dilutions of the lysate were plated on 7H11 agar plates. The CFU counts were recorded after 15 days.

### Staining for Flow Cytometry for Cytokine Analysis and Cell Surface Markers

Following antibodies were acquired: Anti-CD11b (clone: M1/70)-APC, anti-CD11c (clone: N418)-FITC, anti-TNF-α (clone: MP6-XT22)-PE, anti-IL-6 (clone: MPS-20F3)-PE, anti-IL-12 (clone: C15.6)-PE, anti-IL-22 (clone: Poly5164)-PE, anti-IL-10 (clone: JES5-16E3)-PE, anti TGFβ (clone: TW7 -16B4 ) PE, IL1α (clone: ALF-161) PE,(all from Biolegend, USA),CD80 (clone:1610A1) PE, MHCII (clone: M5/114.15.2) PE,CD86 (clone: P03.1) PE, CD1 4(clone: sa2-8) PE, PD1 (clone: J43) PE, CD54 (clone: YN1/1.7.4) (PE all from eBiosciences,USA and PI from BD pharmingen™, USA).

Infected murine intraperitoneal macrophages (infected as described above) were cultured for 48 hours and used for surface staining. For intracellular staining 1×10^6^ cells were cultured per well in 24-well plates (Nunc, USA) and activated with 50 ng/ml phorbol 12-myristate 13-acetate (PMA) and 500 ng/ml ionomycin (Sigma, USA) overnight, and 10 µg/ml Brefeldin A (eBiosciences, USA) was added during the last 6 hours of culture. Cells were washed twice with PBS and stained with antibodies directed against surface markers. After staining, cells were washed again with PBS and fixed with 100 µl fixation buffer (eBiosciences, USA) for 30 minutes, then re-suspended in 200 µl permeabilization buffer (eBiosciences, USA) and stained with fluorescent labelled anti-cytokine antibodies. Fluorescence intensity of fluorochrome-labelled cells was measured by flow cytometry (FACS Canto™ II, BD Biosciences, USA). Cell viability dye (PI) was added to the cells 15 minutes before analyzing the cells by flow cytometry. FACS Diva was used for acquiring the cells and final data analysis was performed by Flow Jo (Tree star, USA).

### Confocal Microscopic Studies

For confocal microscopic studies, the infection was carried out as above with minor changes. The mycobacterial cells were FITC-labeled and washed before using them for infecting macrophages seeded on coverslips. The infection was done at an MOI of 5. FITC (Sigma, USA) binds non-specifically to the mycobacterial cell surface and stains them green. The coverslips were then washed with PBS and fixed with 2.5% paraformaldehyde for 20 minutes followed by a wash with PBS. The cells were permeabilized by treatment with 0.1% NP-40. The coverslips were then stained with antibodies against LAMP-1 (anti-mouse CD107a, BD Pharmingen™, USA), followed by Alexa Fluor 594 goat anti-rat antibodies (Invitrogen, USA). The coverslips were mounted on glass slides using moviol and observed using 488 (green) and 560 (red) nm lasers on Nikon A1R microscope.

## Results

### Expression of ARPC4 Significantly Affects Mtb Growth in Axenic Culture

H37Rv Mtb was transformed with ARPC4pVV16 and 3 individual colonies were picked and analyzed by plasmid mini-prep followed by PCR for the presence of the plasmid DNA. Expression of ARPC4 in Mtb was confirmed by checking the transcription of ARPC4 mRNA; a clear band was seen in the H37Rv/ARPC4 sample.

**Figure 1:**
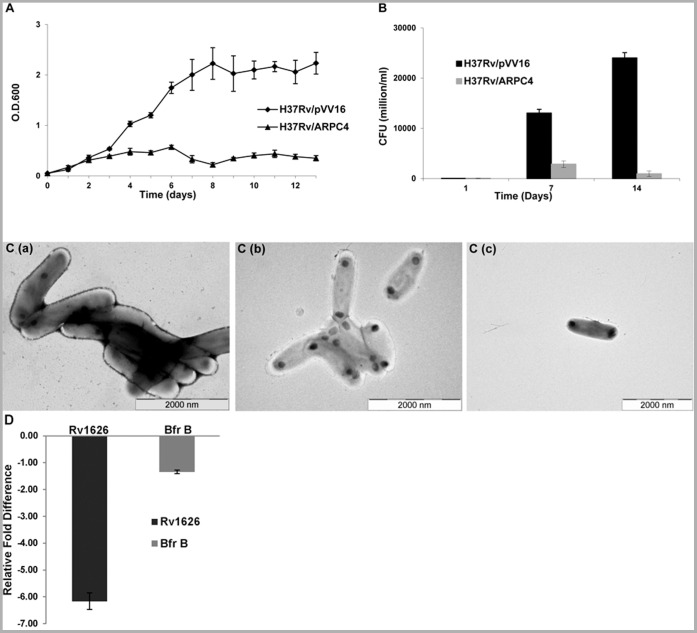
Effect of ARPC4 on the growth of *M. tuberculosis*. A. An individual colony each from mycobacteria harbouring either ARPC4pVV16 (H37Rv/ARPC4) or pVV16 empty vector (H37Rv/pVV16) was picked for growth curve analysis. Optical density of both cultures was measured at 600 nm. Mean±s.d values are plotted against time (in Days). A similar growth curve pattern was observed each time the experiment was repeated. Triangular data points represent OD_600_ values of H37Rv/pVV16 samples (control); circles represent OD_600_ values of H37Rv/ARPC4 samples. **B.** Cultures were grown in 7H9 medium at 37°C with shaking under axenic conditions. The figure shows result of CFU counts of H37Rv/ARPC4 and H37Rv/pVV16 at days 0, 7 and 14. A significant decline in the survival of mycobacteria carrying ARPC4pVV16 was observed in comparison to the vector control. **C.** Effect of ARPC4 expression on cell morphology growth of *M. tuberculosis*. Transmission electron micrographs of H37Rv (panel a) and mycobacterial cells harboring ARPC4pVV16 (H37Rv/ARPC4) (panels b and c). **D.** Relative fold change in the transcript levels of *rv1626* and *bfrB* genes. The *rv1626* gene (dark grey) was found to be significantly down-regulated (6-fold) in H37Rv/ARPC4 cells when compared with the H37Rv mycobacterial cells, whereas the unrelated *bfrB* gene (light grey) expression was not much perturbed. The 16S rRNA gene was used as the normalization internal control. Experiment was performed in triplicates.

To study the effect of ARPC4 on general growth of Mtb, a growth curve analysis was performed wherein optical density of H37Rv/ARPC4 and H37Rv/pVV16 (empty vector) cultures was measured after every 24 hours and plotted against time (in days). The experiment was repeated thrice. Similar pattern was observed each time. It was found that the Mtb harbouring ARPC4pVV16 showed severe retardation in growth when compared with the vector control (figure 1A). The difference – eight to ten-folds – became evident as soon as the cells entered log phase and lasted till stationary phase. As a confirmation of the growth-curve study, a CFU count was carried out. At three different time points, i.e., at day 0, 7 and 14, the culture was removed under sterile conditions and four different dilutions were plated on 7H11 agar plates. Mycobacterial colonies were counted after 10–15 days and the number was plotted against time (in days). A significant reduction in the mycobacterial CFUs was observed in the culture of H37Rv/ARPC4, in comparison to that of H37Rv/pVV16 (figure 1B), thereby corroborating the growth-curve study.

### Expression of ARPC4 Protein in Mtb Alters the Cell Morphology

The deleterious effects of ARPC4 expression in Mtb, as observed by the growth-curve and CFU counts, led us to investigate its effect on cell morphology. TEM studies showed that, compared with the control (H37Rv) sample, H37Rv/ARPC4 cells are smaller in size and show shedding and thinning of the outer coat of the cell (figure 1C b and c). The mycolic acid layer is non-contiguous and is coming off the cell surface as thread-like structures, whereas the control H37Rv cells have a thick and continuous cell coat (figure 1C a).

### Expression of ARPC4 Down-regulates *rv1626* Gene Expression

To underline the cause of the effect, namely the interaction of ARPC4 and Rv1626 proteins, real-time PCR was performed wherein *rv1626* transcript levels of H37Rv/ARPC4 were compared with those in control H37Rv cells. 16S rRNA gene was used as the normalization internal control. R*v1626* gene expression was found to be approximately 6-fold down-regulated in H37Rv/ARPC4 cells. An unrelated gene, *bfrB* was also used as a control (figure 1D) which was not affected significantly.

### Recombinant H37Rv/ARPC4 is Incompetent for its Survival in Macrophage

**Figure 2:**
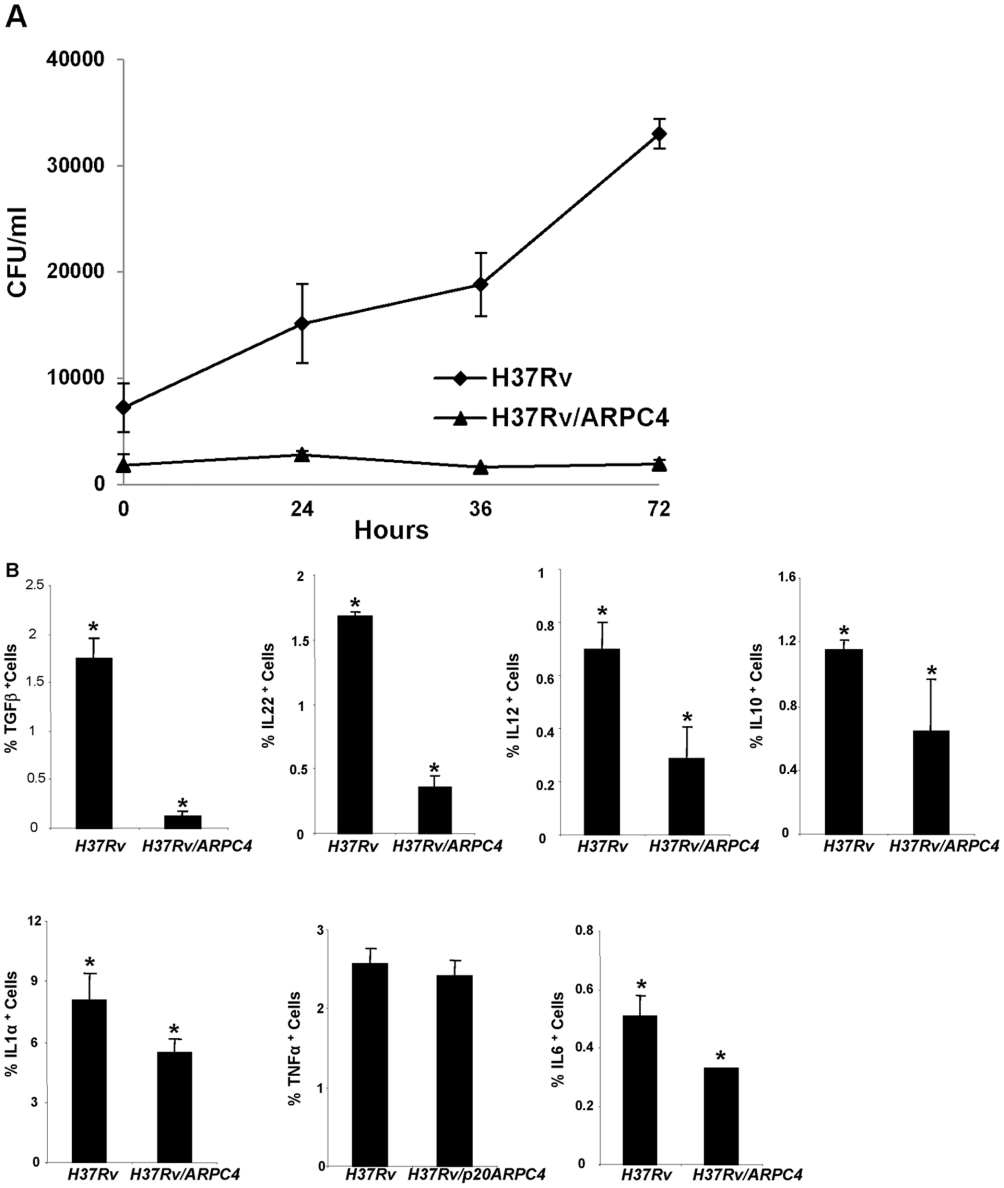
Infection of murine macrophages with H37Rv/ARPC4 mycobacterial strain. **A.** CFU count showing the bacterial load in macrophages infected with H37Rv/ARPC4 and wild-type H37Rv. The infected macrophages were lysed at 0, 24, 48 and 72 hour time-points post-infection and four dilutions of the released mycobacterial cells were plated on 7H11 agar plates. CFU were counted and recorded after 15 days of plating. **B.** The plots show the cytokine milieu in macrophages. Macrophages were cultured and activated with 50 ng/ml phorbol 12-myristate 13-acetate (PMA) and 500 ng/ml ionomycin overnight, and 10 µg/ml Brefeldin A was added during the last 6 hours of culture. Cells were then intracellularly stained with anti-IL22, -TGFβ, -IL10, -IL12, -IL6, TNFα, and IL1α antibodies. Cells were acquired by flow cytometer. Data are shown as mean±STDEV and Student’s t-test was applied for estimating significance between two groups and the * denotes p<0.01.

**Figure 3:**
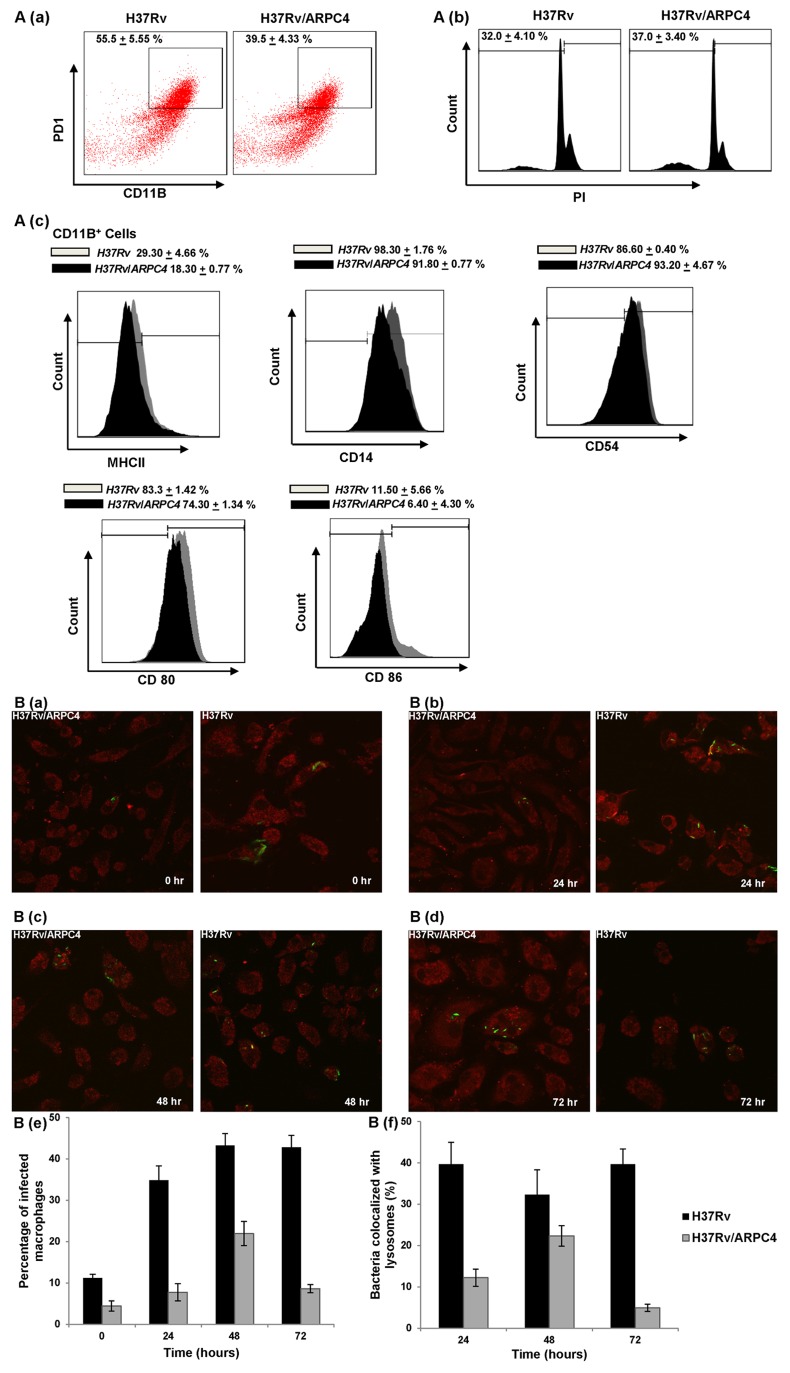
Activation of infected macrophages and inter-compartmental localization of mycobacteria within A. The plots depict reduced activation of H37RV/ARPC4 infected macrophages. **a)** Percentage of cells expressing PD1 among CD11B+ cells is shown in the dot plots with mean±STDEV. Intraperitoneal macrophages isolated from mice were cultured and, 48 hours post-infection with H37Rv and H37Rv/ARPC4 strains at 10 MOI, were surface-stained with anti-CD11B and PD1 antibodies and samples were acquired by flow cytometry. Data shown here are representative of three independent experiments. **b)** Cell death in H37Rv and H37Rv/ARPC4 Mtb infected macrophages. Intraperitoneal macrophages isolated from mice were cultured and, 48 hours post-infection with H37Rv and H37Rv/ARPC4 strains in 1∶10 ratio, were surface-stained with anti-CD11B, CD11C antibodies followed by PI staining for 20 minutes prior to acquisition by flow cytometry to assess cell death in infected macrophages. The percentage of cells expressing PI among CD11B±cells is shown with mean±STDEV. Data shown here are representative of three independent experiments. **c)** Expression of macrophage activation markers. Intraperitoneal macrophages isolated from mice were cultured and, 48 hours post-infection with H37Rv and H37Rv/ARPC4 strains at 10 MOI, were surface-stained with anti-CD11B, CD11C, MHCII, CD14, CD54, CD80, CD86 and CD69 antibodies and samples were acquired by flow cytometry. CD11B+ cells were gated for expression of MHCII, CD14, CD54, CD80 and CD86 and the percentage of cells expressing these markers are shown in the histogram overlay plots with mean±STDEV. Data shown here are representative of three independent experiments. **B.** Confocal Microscopy images showing that H37Rv/ARPC4 mycobacterial strain is less infectious and persistent in macrophage. FITC-labelled mycobacterial cells (green) were used for infecting macrophages (red) at an MOI of 5. At specific time-points, the cells were fixed, permeabilized and stained with anti-LAMP1 antibodies, followed by Alexa Fluor 594 goat anti-rat antibodies. (a)- (d) shows the infected macrophage cells at 0, 24, 48 and 72 hours' time-points post-infection, respectively. (e) and (f) are the graphical representation of the percentage of macrophage cells infected with FITC-labeled mycobacteria at different time-points and the percentage of bacteria localized in lysosomes in the infected cells, respectively. Experiments were repeated thrice and similar results were obtained.

Having witnessed the difference in growth *in vitro*, as well as abrupt morphological changes, we sought to determine the survival of recombinant H37Rv/ARPC4 in macrophages, the natural host for Mtb. We observed that H37Rv/ARPC4 cleared rapidly, whereas wild type Mtb continued to grow (figure 2A). To further test whether rapid clearance is due to enhanced macrophage activation, we determined cytokine production in infected macrophages. We found that H37Rv/ARPC4 infected macrophages produce significantly less cytokines than those macrophages infected with wild type Mtb (figure 2B). To further ensure that macrophages are indeed not activated, we determined expression of MHCII, CD14, CD54, CD80 and CD86. As expected we noticed that these markers are not up-regulated, while wild-type Mtb infected macrophages resulted in the up-regulation of these molecules (figure 3A). These observations indicate that faster clearance of the mutant was not due to the altered macrophage activation, but rather its intrinsic inability to adapt to host cellular environment.

### Confocal Microscopy shows Inhibited Translocation of H37Rv/ARPC4 to Phagolysome

To further study of mechanism of faster clearance of H37Rv/ARPC4 in macrophages, we analysed intra-compartmental localization by confocal microscopy. As expected number of H37Rv/ARPC4 was found to be dramatically less within macrophages as compared to that of wild type H37Rv strain in all the time-points measured (figure 3B a–d). The same is represented graphically as a comparison of the percentage of infected macrophage cells for the total number of macrophages counted (figure 3B e). We further noticed that very few H37Rv/ARPC4 cells are within phago-lysosomal compartment (figure 3B b–d). A quantitative measure is presented (figure 3B f).

### Rv1626 Interacts with ARPC4 Subunit of Arp2/3 Protein

**Figure 4:**
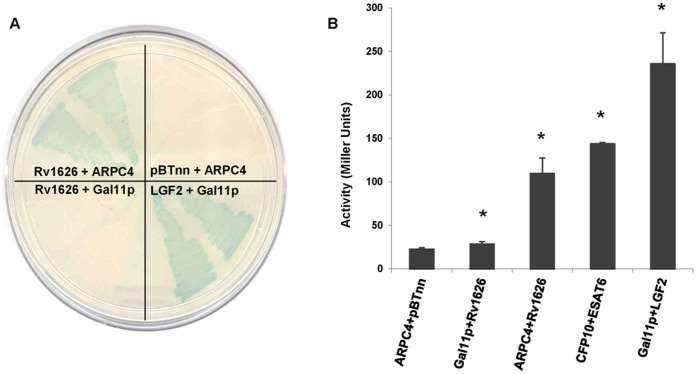
Rv1626-ARPC4 protein-protein interaction by bacterial two-hybrid. **A.** Bacterial two-hybrid plate showing Rv1626 and ARPC4 interaction where blue colored colonies show interaction between the bait and target proteins. BacterioMatch two-hybrid *E. coli* reporter strain was co-transformed with Rv1626-pBTnn+ARPC4-pTRGnn; pBTnn+ARPC4-pTRGnn (negative control); LGF2-pBT+Gal11p-pTRG (positive control); and Rv1626-pBTnn+Gal11p-pTRG (negative control) plasmids. Two individual colonies from each co-transformant were patched on X-gal indicator plate. **B.** Histogram of liquid β-galactosidase assay for quantitative estimation of interaction strength of Rv1626 and ARPC4 compared to that with positive and negative controls. Co-transformants ARPC4-pTRGnn+pBTnn and Gal11p-pTRG+Rv1626-pBTnn were taken as the negative controls; CFP10-pTRGnn+ESAT6-pBTnn and Gal11p-pTRG+LGF2-pBT were taken as positive controls; and these were used to compare the β-galactosidase activity of co-transformant ARPC4-pTRGnn+Rv1626-pBTnn. Enzyme activity is a direct indication of the strength of interaction of the bait and target proteins and, is expressed in terms of Miller Units. All interactions were found to be statistically significant by Student’s t-test (*, P<0.05).

In order to elucidate the role of Rv1626, a bacterial two-hybrid system was used to fish out interaction partner(s) of the secretory protein from the human lung cDNA library. A distinctly blue-colored colony was isolated. Sequencing result followed by a BLAST search identified the interacting partner as the ARPC4 subunit of the Arp2/3 protein complex. The interaction was confirmed by several rounds of plasmid segregation followed by co-transformation and re-cloning of ARPC4 in pTRGnn vector followed by re-cotransformation (figure 4A).

For a quantitative estimation of the strength of interaction of Rv1626 and ARPC4, liquid β-galactosidase assay was performed. The enzyme activity in case of Rv1626 and ARPC4 interaction was found to be comparable to that of a known mycobacterial protein-protein interaction, that of CFP10 and ESAT6 proteins which are both mycobacterial proteins and are well documented to form a tight heterodimer [23], showing that the interaction of Rv1626 and ARPC4 is strong (figure 4B).

### *In vitro* Interaction of Rv1626 and ARPC4 Confirmed by Dot Far-Western Analyses

**Figure 5:**
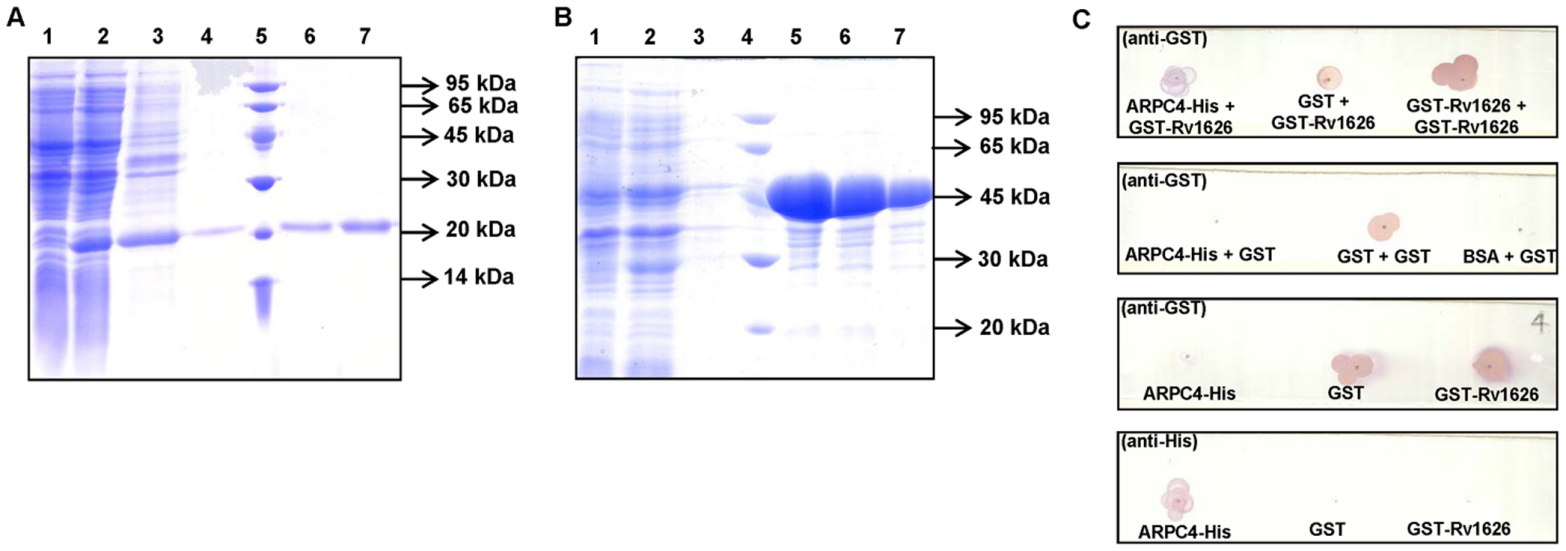
Rv1626-ARPC4 protein-protein interaction *in vitro*. **A.** Coomassie-stained purification gel of ARPC4-His_6X_. ARPC4-His_6X_ protein was purified under denaturing conditions on a Ni-NTA column. 15% SDS polyacrylamide gel showing purified C-terminal His-tagged ARPC4 protein, stained with coomassie blue. Lane 1: uninduced cell lysate, Lane 2: induced cell lysate, Lane 3: supernatant fraction of cell lysate (load), Lane 4: final wash, Lane 5: low molecular weight protein marker (the sizes, in kDa, are indicated on the left of the panel), Lane 6–7: purified protein elution fractions. **B.** Coomassie-stained purification gel of GST-Rv1626 proteins GST-Rv1626 protein was purified under native conditions on a Glutathione Sepharose-4B resin column. 15% SDS polyacrylamide gel showing the purified GST-Rv1626 protein, stained with coomassie blue. Lane 1: uninduced cell lysate, Lane 2: induced cell lysate, Lane 3–7 (except 4) are fractions collected from the column; Lane 3: wash fraction, Lane 4: low molecular weight protein marker (the sizes, in kDa, are indicated on the left of the panel), Lane 5–7: purified protein elution fractions. **C.** Dot Far-Western Blots showing *in vitro* protein-protein interactions. 1 µg each of purified ARPC4-His_6X_, GST, GST-Rv1626 or BSA proteins were immobilized on nitrocellulose strips that were incubated overnight in 1 µg/mL solution of the either GST-Rv1626 or GST protein. The strips were then developed using the antibody indicated in the parenthesis.

The ARPC4:Rv1626 interaction was confirmed *in vitro* by Dot Far-Western experiments. For this, ARPC4 was expressed as a C-terminal His-tagged protein and purified using a Ni-NTA agarose column (figure 5A), while Rv1626 was expressed as an N-terminal GST-tagged protein, GST-Rv1626, and purified using Glutathione Sepharose-4B resin (figure 5B). ARPC4 was immobilized onto a strip of nitrocellulose membrane and the strip incubated with GST-Rv1626. When probed with anti-GST antibodies, Rv1626 could be detected over the ARPC4 spot, confirming the interaction between ARPC4 and Rv1626 *in vitro*. False or erroneous interaction because of the 26 kDa GST tag was ruled out by replication of the experiment wherein incubation of the strip was carried out with GST. GST could not be detected on this control strip. All the positive control dots of immobilized GST/GST-Rv1626 proteins were detectable on both the strips, nevertheless (figure 5C).

### Rv1626 Interacts with Arp2/3 Complex

**Figure 6:**
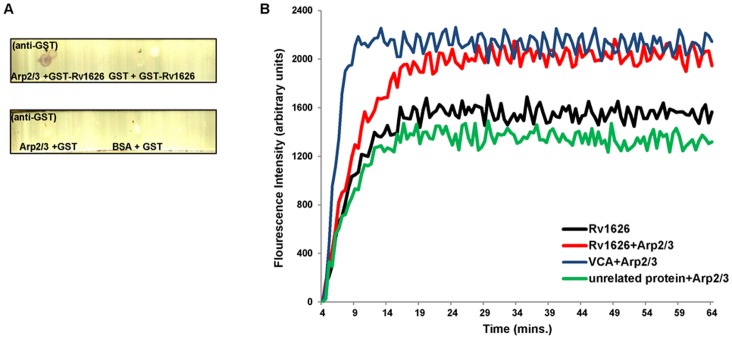
Rv1626-Arp2/3 protein-protein interaction. **A.** Dot Far-Western Blots showing *in vitro* protein-protein interaction between Arp2/3 and Rv1626. 1 µg each of Arp2/3 and BSA proteins were immobilized and the strips incubated overnight in 1 µg/mL solution of the either GST-Rv1626 or GST protein followed by development using anti-GST antibody. **B.** Rv1626 interaction with Arp2/3 enhances Actin Polymerization. Polymerization of G-actin to F-actin leads to an increase in the fluorescence intensity of pyrene-labelled actin, plotted here as a function of time. The figure shows the effect of Rv1626 protein on the rate of actin polymerization. Rv1626 (500 nM alone, black); Arp2/3 (10 nM) in the presence of Rv1626 (500 nM, red), VCA domain (400 nM, blue), and an unrelated protein (500 nM, green).

The ARPC4 protein is a subunit of the large 224 kDa seven subunit Arp2/3 complex. Therefore, we wanted to further investigate whether the interaction of Rv1626 is confined only to the ARPC4 subunit or whether it is also able to interact with the entire Arp2/3 complex as well. For this, Rv1626 protein was purified according to the method described earlier [9] and Dot Far-Western analyses were performed. In this experiment, when Arp2/3 and BSA were immobilized on a nitrocellulose membrane strip and the strip was incubated with GST-Rv1626, upon probing with anti-GST antibodies, GST-Rv1626 was detected on the dot corresponding to Arp2/3 while no protein was detected bound to BSA. A control strip was incubated with GST just as above; no protein-protein interaction was detected on this strip (figure 6A).

Employing traditional pull-down and co-immunoprecipitation assays for confirming the *in vitro* interaction of Rv1626 with the entire Arp2/3 complex were unsuccessful. It could be that the interaction of Rv1626 with ARPC4 in context of the entire seven subunit Arp2/3 protein complex may be altered, even transient, and therefore undetectable by conventional methods, given that these *in vitro* tools depend primarily on the high stability and strength of the interaction under study [24].

### Rv1626-Arp2/3 Interaction Enhances Actin Polymerization Rate

It has been shown for many pathogenic proteins that their interaction with Arp2/3 increases its efficiency of actin polymerization. In the assay, the enhanced fluorescence resulting from pyrene Globular- or G-actin (monomer) polymerizing to form pyrene F-actin (Filamentous actin) is measured using a fluorimeter to follow the polymerization over time. We decided to investigate whether Rv1626 is able to enhance actin polymerization in the presence of Arp2/3. Polymerization of actin was monitored in presence of both Arp2/3 and Rv1626, alone and in combination (figure 6B). A buffer control (PBS), a positive control (GST-tagged VCA (Verprolin, cofilin, acidic) domain of human WASP protein) and a negative control (unrelated protein) were also run. The rate of polymerization of actin in PBS was observed to be nearly the same as in presence of the unrelated protein with Arp2/3 and both these were only marginally lower than the polymerization induced by Arp2/3 alone. This confirms the fact that Arp2/3 alone cannot polymerize actin very efficiently without being activated by an NPF (nucleation promoting factor). On the other hand, actin polymerization was found to be much higher in presence of both Rv1626 and Arp2/3 proteins when in combination. Actin polymerization in the positive control – VCA domain with Arp2/3– was found to be far higher than that with both proteins alone.

## Discussion

Expression of ARPC4 inside Mtb causes severe growth retardation, comparable to that caused by Granulysin on Mtb cultures, or indeed of Mtb strains where essential genes like *glnR* are knocked out [25]. The 6-fold down-regulation of *rv1626* gene expression in ARPC4-expressing H37Rv cells and the extent of growth retardation because of it projects Rv1626 as a potential drug target. As discussed earlier, structural predictions indicate Rv1626 as a possible transcription anti-terminator. It is quite likely that, by virtue of its interaction with Rv1626, ARPC4 sequesters Rv1626 and makes it unavailable for the anti-termination function. This unavailability may also affect the expression levels of a multitude of genes. This could be further investigated by DNA microarray experiments that are currently underway in our laboratory.

The TEM results show significant differences between the morphology of wild type and ARPC4-expressing strains, for example the extensive shedding of the cell wall, an outcome that could result in significant loss of cell surface proteins. These proteins play protective role for the bacterium to mask it from the host defence system [26]. To corroborate this, macrophage infection studies were carried out which indicated H37Rv/ARPC4 to have greatly lost its infectivity as well as persistence in macrophages. It is being cleared off from the macrophages with such rapidity that cytokine production is not being triggered and the host cells are not activated to mount any immunogenic response. Furthermore, the confocal microscopy results show that H37Rv/ARPC4 bacteria are not able to translocate to the lysosomal compartment. This indicates that although the cytokine-stimulated macrophage phagocytic pathway is not being triggered, the ARPC4-expressing mycobacteria are in itself incapable of survival and persistence within the macrophages.

Mtb has approximately 4000 protein coding genes (JCVI-CMR: *Mycobacterium tuberculosis CDC1551* Genome). For a pathogen this is a rather conservative number, as a pathogenic bacteria has to take care not only of its own cellular functions but also has to devise ways to tackle the defence mechanisms of the host. Recent studies are bringing forth the fact that this apparent shortcoming is compensated through many of its proteins performing a multitude of functions, i.e. the proteins are multi-functional or are ‘moon-lighting’ proteins.

Predictions based on the reported structure indicate Rv1626 protein to be a phosphorylation-dependent transcription anti-terminator [9]. Based on our studies, an additional possible role for Rv1626, that of aiding Mtb to establish infection in macrophages, may be worthy of further investigation. Our experiments show that mycobacterial Rv1626 interacts with the ARPC4 subunit of Arp2/3, and in turn also with the entire complex itself, a complex that plays a vital role in the maintenance of the mammalian cell cytoskeleton. Arp2/3 is an actin polymerizing protein complex and is involved in all the motile activities of the cell that require dynamic actin filament assembly, like cell migration, endocytosis, vesicle trafficking, cytokinesis etc [27]. In cells Arp2/3 is the main factor that regulates the state of actin. In actin polymerization assay it was seen that the rate of spontaneous condensation actin to form filamentous actin was nearly the same as when in the presence of Arp2/3. This confirms the requirement of an activator of Arp2/3 to bring about significant polymerization. Actin polymerization in the presence of both Rv1626 and Arp2/3 as a combination was seen to be much higher than in presence of both the proteins separately. The ability of Mtb to alter host cell cytoskeleton has been reported previously [28]. In the case of *Mycobacterium marinum*, it has been reported that the bacterium not only breaks free from the phagosome and enters the cytoplasm of the infected macrophages but also uses actin-based motility for direct cell to cell spread by inducing actin polymerization through recruitment of host cytoskeletal factors viz. Arp2/3 and WASP (Wiskott Aldrich Syndrome Protein). The mycobacterial factor(s) involved in this, though, still remain unknown [29], [30]. In case of *M. tuberculosis*, the long-held belief of the pathogen’s confinement in phagosomes was challenged by reports clearly showing Mtb free in the cytoplasm of the infected cell and capable of direct cell to cell spread in tissue culture [31]–[34]. The mycobacterial factors involved in this function are yet to be elucidated. The interaction of mycobacterial Rv1626 with human Arp2/3, could form the premise of this missing link.

Moreover, the discovery that a natural protein, i.e. ARPC4, whose crystal structure is already known, interacts strongly with an essential mycobacterial protein, could become a starting point for peptidomimetic studies with the singular aim of finding anti-TB molecules. Structural analyses could shed light on the interaction interface of the two proteins, making ARPC4 a convenient peptidomimetic template for further studies. The search for newer drugs against this dreaded pathogen is an urgent one, and with the sparse success achieved through compound library-based approaches, the area of peptidomimetics has over recent years gained added importance. A closer inspection of the ARPC4-Rv1626 interaction may become a starting point for creating a library of small molecules that are able to inhibit the functions of Rv1626.
